# High-Temperature Wetting Behavior and Adhesion Mechanism of Cryolite-Based Molten Salt on SiC Refractory Substrate

**DOI:** 10.3390/ma18071428

**Published:** 2025-03-24

**Authors:** Yuxi Feng, Wandong Cheng, Zhiyuan Rui, Haobo Sun, Xin Lyu, Yun Dong

**Affiliations:** 1 School of Mechanical and Electrical Engineering, Lanzhou University of Technology, Lanzhou 730050, China; lutfyx@163.com (Y.F.);; 2 Engineering Research Center of New Nonferrous Metallurgy Equipment, Ministry of Education, Lanzhou University of Technology, Lanzhou 730050, China

**Keywords:** wetting and adhesion behaviors, cryolite-based molten salt, SiC substrate, work of adhesion

## Abstract

The problem of the adhesion of aluminum slag to the inner wall of a vacuum ladle is essential but has not been addressed. Using a high-temperature wettability experimental setup, this paper investigates the mechanism of interfacial wettability, adhesion, and penetration between Na_3_AlF_6_-Al_2_O_3_-CaF_2_ cryolite-based molten salt and SiC refractory substrate. The composition of the slag was determined based on the slag obtained in the transfer ladle during the aluminum electrolysis process. We mainly study the effects of different Al_2_O_3_ contents in cryolite-based molten salt and temperatures on the contact angle and surface tension. The results indicate that as the Al_2_O_3_ content in the slag increases, the contact angle decreases, enhancing the slag’s wettability on the SiC substrate. Additionally, a higher Al_2_O_3_ content leads to higher slag melting temperatures and surface tension, which improves the slag mobility and enhances the mass transfer and diffusion capabilities of molecules or ions within the slag. The work of adhesion, calculated using the Mills model, also increases with the increasing Al_2_O_3_ content. The increased Al_2_O_3_ concentration activates the activity of Na_3_AlF_6_ in the slag, facilitating the dissolution reactions and improving the wettability between the slag and SiC. Moreover, the wetting behavior of the Na_3_AlF_6_-Al_2_O_3_-CaF_2_ slag is primarily influenced by the initial Al_2_O_3_ content and its compositional changes. The results show that the slag penetration resistance and mechanical erosion resistance of the ladle lining can be improved by using an SiC-based refractory with an optimized Al_2_O_3_ content. This will have important guiding significance for the development, design, and application of inner wall materials for aluminum electrolysis industrial vacuum ladles.

## 1. Introduction

In the industrial process of aluminum electrolysis, a significant operational challenge arises during the transfer of molten primary aluminum from vacuum ladles to mixing furnaces via electrolysis cells. During this critical phase, a significant amount of aluminum slag containing various production raw materials forms strong adhesive bonds with the refractory lining of the vacuum ladles. This problematic adhesion phenomenon not only compromises the structural integrity of the ladle lining but also significantly shortens the operational life of these essential metallurgical vessels. Consequently, this technical problem leads to increased maintenance frequency and significant operating costs in aluminum production facilities [1,2,3]. Given these significant economic and technical implications, there is an urgent need to develop effective solutions to mitigate the interfacial adhesion between aluminum slag and the refractory materials used in vacuum ladle linings.

The interaction between slag, aluminum liquid, and refractory is a typical and complex multiphase flow behavior in the high-temperature zone of an electrolyzer. The wetting behavior between these phases is the most fundamental feature that determines their interactions. Aluminum slag, which is crucial in metallurgy, interacts with the melt. The slag interfacial properties impact the mobility, material migration, and wettability of metal, refractory, and slag components [4,5,6,7]. Few studies have been conducted on the wettability of aluminum slag and SiC refractories. Studying interfacial wettability is essential for understanding and controlling interfacial phenomena and developing new electrolytic aluminum ladle devices.

The inner wall material of the vacuum ladle is mainly composed of SiC- and Al_2_O_3_-based refractory casting materials. The erosion of the inner wall of the refractory is related to chemical reactions and physical processes. SiC has excellent properties, such as high thermal conductivity, low coefficient of thermal expansion, high melting point (2700 °C), poor wettability to slag, high hardness, and resistance to chemical erosion. Therefore, it is widely used in the abrasives, metallurgy, energy, and chemical industries [8]. During the transit of high-temperature aluminum liquids, the wettability between the slag and SiC can change due to variations in temperature and composition. The sessile droplet method can be used to study the wettability between slag and refractory. Slag droplets quickly penetrate and wet the SiC substrate through its pores and grain boundaries, which were initially considered non-reactive [9,10]. During prolonged high-temperature operations, refractory constituents progressively dissolve into the molten slag system, refs. [11,12] initiating complex physicochemical interactions. This dissolution process enhances mass transport phenomena within the slag phase, ultimately driving phase transformation through interfacial chemical reactions that generate thermodynamically stable crystalline/amorphous products [13,14]. Notably, Monaghan et al. [15] systematically characterized the dynamic wetting characteristics of multi-component slags on alumina-based ceramic substrates using the high-temperature sessile drop method. Their experimental data revealed two distinct wetting stages: an initial rapid decrease in the slag-refractory contact angle (*θ*) within the first 10 s of interaction, followed by stabilization into a quasi-equilibrium wetting state. Furthermore, this study demonstrated a significant inverse correlation between slag acidity and wettability, with *θ* values decreasing systematically as the basicity index increased. Beyond compositional factors, slag viscosity is a critical rheological parameter governing interfacial phenomena. Recent studies [16,17,18,19,20,21] have established that viscosity not only dictates capillary infiltration kinetics but also directly modulates the kinetic progression of slag-refractory interactions, thereby serving as a crucial determinant of refractory degradation rates and operational lifespans in high-temperature metallurgical processes. For the slag of the ironmaking system, Zuo et al. [22] explored the effect of slag viscosity on the wettability behavior of refractory materials and showed that when the mass fraction of MnO increases, the slag’s melting temperature and viscosity decrease simultaneously, and its wettability gradually increases. Consequently, the high-temperature wettability of the refractory materials increases, exacerbating the erosion of the slag on the refractory materials. Xu et al. [23] studied the effect of slag viscosity on refractory substrates. They found that increasing the content of MnO in the CaO-SiO_2_-MgO-Al_2_O_3_-Cr_2_O_3_ slag results in a gradual decrease in slag viscosity and melting point, and the erosion of the slag on the carbon composite refractory is the result of infiltration, melting, and the reaction of the slag-refractory. Yoon et al. [24] investigated the effect of slag composition on the viscosity and melting temperature of the CaO-Al_2_O_3_-SiO_2_-MgO tetrameric slag and its wetting behavior on Mg-rich spinel substrates and Al-rich spinel substrates. The permeability of the slag increased with the increase in MgO content in spinel, and the resistance of the refractory to slag erosion increased with the increase in Al_2_O_3_ content in spinel. Sahajwalla et al. [25] investigated the wettability of slag and demonstrated that increasing the iron oxide content and temperature enhances wettability. The authors further observed that at elevated temperatures, gas formation due to chemical reactions leads to slag foaming and fluctuations in the contact angle. Compared with prior studies, this work employs a SiC substrate, thereby minimizing extraneous variables and improving experimental accuracy. Under high-temperature conditions, the sessile drop method is commonly utilized to assess the wettability of molten slag interfaces [26]. Recent studies have highlighted the importance of advanced refractory materials, such as Refractory High-Entropy Alloys (RHEAs), Refractory High-Entropy Composites (RHE-Cs), and Refractory High-Entropy Ceramics (RHE-Ce) due to their superior properties compared with those of traditional alloys. These materials are known for their high melting points, high-temperature resistance, and high corrosion and wear resilience. Our study aligns with these advancements by focusing on the optimization of SiC-based refractory materials widely used in the aluminum electrolysis industry. The findings from our research will provide valuable insights into how the composition and properties of these materials can be further optimized to enhance their performance in high-temperature environments.

While previous studies have explored the wetting behavior of molten aluminum on SiC and the corrosion mechanisms of slag on refractory materials, a detailed investigation of the wettability of aluminum slag on SiC, particularly under conditions relevant to the aluminum electrolysis industry, remains limited. Our study aims to fill this gap by focusing on the effects of varying Al_2_O_3_ concentrations in the slag on its wettability and adhesion properties on SiC. The present study emphasizes the necessity of studying the wettability of aluminum slag and SiC refractory to reveal the mechanism of aluminum slag adhesion on the inner wall of a vacuum ladle. In this paper, the wetting and spreading processes of slag with varying Al_2_O_3_ contents on a SiC substrate are investigated through wetting experiments. The contact angle and melting temperature are measured, with the contact angle determined by the sessile droplet method. The surface tension is calculated using Dorsey’s method. A thorough discussion of the effects of the Al_2_O_3_ composition ratio and temperature on the contact angle, surface tension, and work of adhesion is presented. By theoretical calculations, the mechanism of wetting adhesion of Na_3_AlF_6_-Al_2_O_3_-CaF_2_ slag on the SiC surface is analyzed. Furthermore, the microstructure and phase composition of the slag-SiC interface are examined using optical microscopy and scanning electron microscopy-energy-dispersive spectroscopy (SEM-EDS), with the objective of assessing the permeability of the slag to the SiC surface. The findings of this study can aid in understanding and designing the inner wall of a vacuum ladle with optimal interfacial properties to enhance its resistance to aluminum slag.

## 2. Experimental and Thermodynamic Calculations

### 2.1. Preparation of the Aluminum Slag and SiC Substrate

The experimental procedure involved the selection of a representative Na_3_AlF_6_-Al_2_O_3_-CaF_2_ slag system, with the addition of varying quantities of Al_2_O_3_. The resultant composite slag consisted of analytically pure Na_3_AlF_6_, Al_2_O_3_, and CaF_2_ (Tianjin Zhiyuan Chemical Reagent Co., Ltd., China, Tianjin, China), with the chemical composition details illustrated in Table 1. Five sets of wetting experiment slags were prepared by weighing according to the mass percentages listed in Table 2. The components were then placed in a corundum crucible, thoroughly ground, and combined to form synthetic slag. A mixture of 0.25 g of the synthetic slag and a small quantity of 10% aqueous dextrin solution was thoroughly mixed and subsequently loaded into a steel mold. The mixture was then compressed at 100 MPa for a duration of five minutes to produce a cylindrical sample with dimensions of 8 mm in height and 5 mm in diameter. Subsequent to this, the samples were subjected to oven drying in order to facilitate their use in wetting-related experiments.

**Figure 1:**
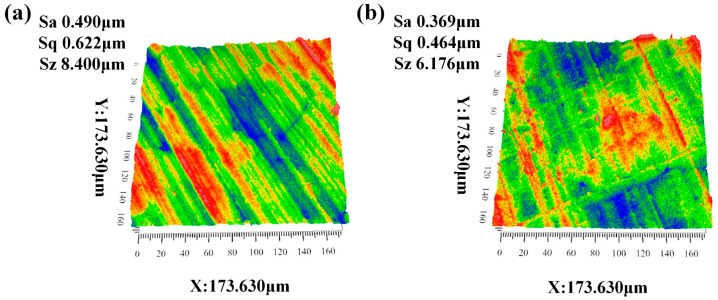
Scanning of the surface features of the SiC substrate. (**a**) Silicon carbide sample No.1; (**b**) Silicon carbide sample No.2.

Refractory substrates made of SiC, measuring 20 mm × 20 mm × 5 mm, were utilized in the experiments. The substrate’s composition was examined, as detailed in Table 3. A ZeGage^TM^ PRO 3D optical profiler (Made by Zygo Corporation in the Middlefield, CT, United States, purchased by Shanghai Representative Office in China, Shanghai, China) from Zygo facilitated the measurement and characterization of the SiC substrate’s surface roughness at the micro-nanometer level, with results presented in Figure 1; the surface roughness of the refractory substrate ranged from Ra = 0.369 to 0.49 μm. Table 4 lists the characteristics of the SiC refractory material. Following the surface treatment, the substrate was cleaned with alcohol using an ultrasonic device, subsequently dried, stored in a dryer, and prepared for wetting experiments.

The experimental setup employed high-purity silicon carbide refractory substrates with nominal dimensions of 20 mm × 20 mm × 5 mm (±0.05 mm tolerance). Comprehensive material characterization, including phase composition (XRD, Rigaku SmartLab, Ricoh Corporation of Japan, Tokyo, Japan) and elemental distribution (EDS, JEOL JSM-7800F, Japan Electronics, Tokyo, Japan), was performed, as detailed in Table 3. Surface topography analysis was conducted using a state-of-the-art ZeGage^TM^ PRO 3D optical profiler (Zygo Corporation, CT, USA) equipped with a 20× Mirau interferometric objective and advanced MetroPro^®^ software (MetroPro8.3.5) for nanoscale surface metrology. The measured surface roughness parameters (Ra = 0.369–0.490 μm, Rz = 2.15–3.08 μm) were statistically analyzed across 25 sampling areas (500 μm × 500 μm each) to ensure measurement reliability, with representative 3D surface morphology profiles presented in Figure 1. Prior to wettability testing, the substrates underwent a rigorous surface preparation protocol: (1) mechanical cleaning: removal of surface contaminants using compressed air; (2) chemical treatment: ultrasonic cleaning (Branson 5800, 40 kHz, Emerson Electric Group, St. Louis, MO, USA) in analytical-grade ethanol (99.9%, Sigma-Aldrich, Sigma Aldrich (Shanghai) Trading Co., Ltd., Shanghai, China) for 15 min at 45 °C; (3) thermal stabilization: drying in a vacuum oven (Binder VD23, 10^−2^ mbar, Germany’s Pentax Group, Neckasulm, Germany) at 120 °C for 2 h; and (4) storage protocol: maintenance in a nitrogen-purged desiccator (RH < 5%, 25 °C) until experimental use. The complete physical and thermochemical properties of the SiC refractory material are listed in Table 4. This comprehensive surface preparation and characterization protocol ensured consistent interfacial conditions for subsequent wettability investigations.

### 2.2. Experimental Methods and Thermodynamic Simulation Calculations

**Figure 2:**
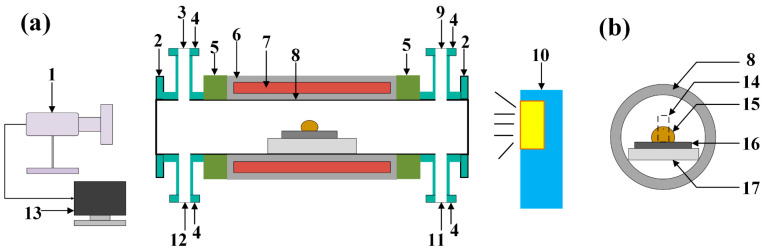
Schematic of the experimental setup (**a**) and side view of the test state in the furnace (**b**). 1—camera; 2—quartz window; 3—gas inlet; 4—sealing cover; 5—flange; 6—refractory; 7—heating element; 8—corundum furnace tube; 9—gas outlet; 10—light source; 11—thermocouple outlet; 12—Pt wire outlet; 13—PC and monitor; 14—cylindrical slag; 15—slag drop; 16—SiC substrate; 17—corundum carrier.

**Figure 3:**
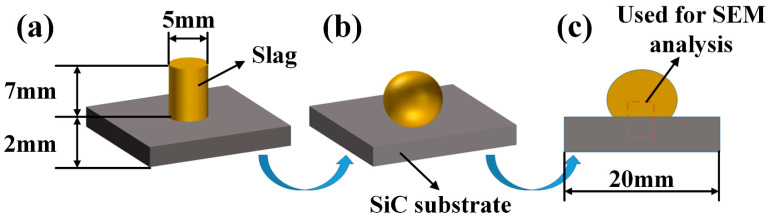
Schematic of the slag and SiC substrate (**a**) before and (**b**) after the slag wetting test, and (**c**) cross-section used for SEM analysis.

The term “wettability” in general, refers to the propensity of a surface to accept or reject liquid. The measurement of this property typically employs the contact angle, which is defined as the angle between the liquid and air interface and the tangent of the surface. The present study investigates the melting and wetting behavior of Na_3_AlF_6_-Al_2_O_3_-CaF_2_ slag with varying alumina contents and SiC refractories. The sessile drop method was employed using a vacuum high-temperature wetting test system (model GSL-1200X-II, HE FEI KE JING MATERIALS TECHNOLOGY Co., Ltd., Hefei, China), as shown in Figure 2. This instrument is capable of measuring the contact angle between the molten slag and the SiC refractory material and observing the melting and wetting processes between them in real-time. The instrument’s specific operating steps can be outlined as follows: (1) The treated SiC refractory was placed on the sample support platform of the high-temperature tube furnace, followed by the placement of simulated slag columns with varying composition ratios at the center of the surface of the SiC refractory substrate. The entire assembly was then positioned horizontally within the designated internal thermostatic zone of the tube furnace, ensuring that the slag columns and the refractory substrate remained in a horizontal state to maintain stability. A schematic diagram is presented in Figure 3a. (2) The experimental furnace was sealed using a quartz glass flange. To expel the gas, the air pressure in the furnace chamber was reduced to −0.8 bar using a mechanical pump, after which 300 mL/min of argon gas (99.99% purity) was introduced into the furnace tube via a mass flow meter. It should be noted that all experiments were carried out in high-purity argon gas. (3) Subsequent to a thorough washing of the furnace, the desired temperature was increased in 5 °C increments every 15 min according to a predetermined temperature regime, and the temperature was held constant for a duration of 15 min. The cooling sequence then commenced at the same temperature rate. (4) During the experimental period, the onset of the slag column change on the refractory substrate was taken as the starting point in time, and no further change was observed, marking the end of the experiment. The wetting process was recorded by means of a high-resolution digital video camera at a frame rate of 12 pictures per minute in order to document the alterations in the droplet profile, reflecting the changes in the wetting angle during the melting-wetting process. Figure 3b presents a schematic representation of the aluminum slag and SiC refractory following the slag wetting test. (5) The experimental data were imported into the computer software ImageJ (lmageJ 1.52i), and the contour data were extracted by utilizing data processing software to vectorize the droplet images. The solid-liquid wetting angle was then measured by fitting and calculating the contour data, with an error within ±2°. Subsequently, the contact angle and wetting radius between the slag and refractory substrate at various temperatures were calculated. Following this, an analysis was conducted on the wetting characteristics. The mean value of two repeated experiments was accepted as the final result. (6) Following the measurement of the contact angle, the slag samples were cooled to room temperature in an oven containing the substrate and then prepared for subsequent analysis. The samples were placed in molds and encapsulated using an epoxy resin. Subsequent to curing the resin, the test samples were cut along the center line using a diamond wire cutter. Thereafter, the resulting cross-sections (illustrated in red as a dashed area within Figure 3c) were subjected to polishing and ultrasonic cleaning. Scanning electron microscopy, in conjunction with energy-dispersive spectroscopy, was employed to analyze the microstructures of the two-phase interfaces and the elemental distributions.

The Gibbs free energy theory and multiphase equilibrium theory can be applied to understand the chemical reactions and mineral behaviors occurring during the melting and wetting of Na_3_AlF_6_-Al_2_O_3_-CaF_2_ slag on the refractory surface [27,28]. Firstly, the equilibrium products of the reaction between 100 g of Na_3_AlF_6_-Al_2_O_3_-CaF_2_ were simulated using the Equilib model in the thermodynamic software FactSage 7.2 of the Gibbs free energy minimization theory in the temperature range of 973–1373 K, with a step size of 10 K, under an argon atmosphere. The wetting mechanism of the Na_3_AlF_6_-Al_2_O_3_-CaF_2_ slag on the refractory was further explained by comparing the FactSage calculations with the experimental results.

## 3. Results and Discussion

### 3.1. Aluminum Slag Melting and the Change of Droplet Morphology

**Figure 4:**
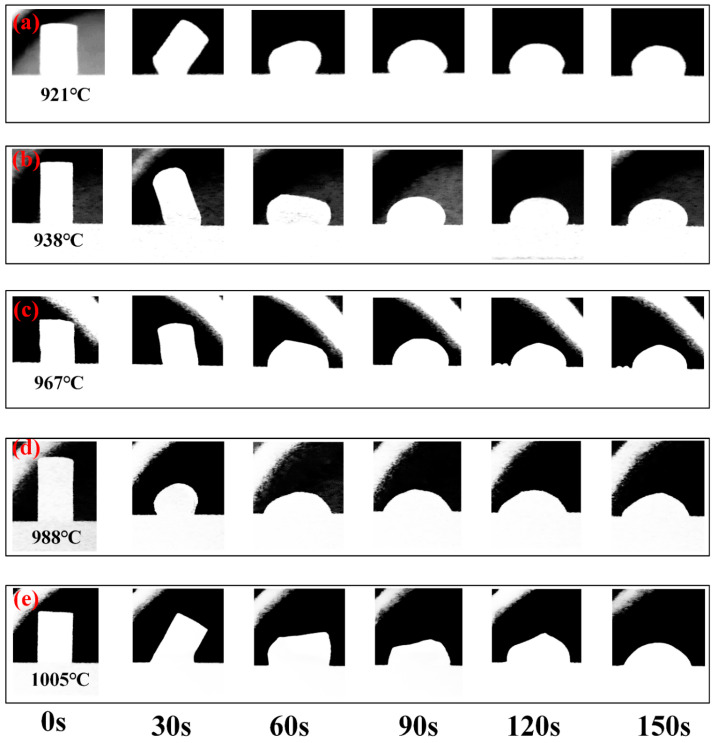
Wetting process between aluminum slag and SiC substrate with different Al_2_O_3_ contents: (**a**) 0 wt% Al_2_O_3_; (**b**) 5 wt% Al_2_O_3_; (**c**) 10 wt% Al_2_O_3_; (**d**) 15 wt% Al_2_O_3_; (**e**) 20 wt% Al_2_O_3_.

A high-speed camera was used to observe the wetting and spreading process of Na_3_AlF_6_-Al_2_O_3_-CaF_2_ slag with different Al_2_O_3_ contents on the SiC refractory. This observation occurred when the temperature was raised to the melting point and maintained for 15 min. The results are shown in Figure 4. The experiment recorded the morphology of the slag, changes in the contact angle, and duration of the wetting process. The collected data were subsequently utilized to facilitate an in-depth investigation into the impact of Al_2_O_3_ composition within the slag on crucial parameters, such as the wetting process of both the slag and the refractory. This comprehensive analysis also encompassed the melt processing of each slag sample and the morphological alterations experienced by the slag droplets as they wetted the SiC substrate across diverse thermal ranges. As shown in Figure 4, with a continuous increase in the temperature of the tube furnace, the slag on the SiC refractory substrate gradually melts. The process begins from the bottom, forming a 5 mm diameter and 8 mm high slag column that spreads and wets the refractory material. As the height of the slag is reduced, the diameter of the bottom surface is increased. The five slag samples are made wet and are distributed on the surface of SiC at different initial melting temperatures and have different wetting rates. As shown in Figure 4, an increase in the experimental temperature and holding time led to the gradual flow and expansion of slag samples with different Al_2_O_3_ contents on the substrate, indicating an enhancement in slag wetting on SiC. The bonding of slag to refractory materials is attributed to the penetration of refractory materials by the components in the slag, slag dissolution reaction, and integrated reaction of the slag and refractory materials. It is, therefore, evident that as the Al_2_O_3_ content increases, the solubility of Al_2_O_3_ concomitantly increases, thus causing an increased amount of SiC refractory material to dissolve in the slag. This phenomenon consequently leads to a decline in the contact angle between slag and the refractory material.

**Figure 5:**
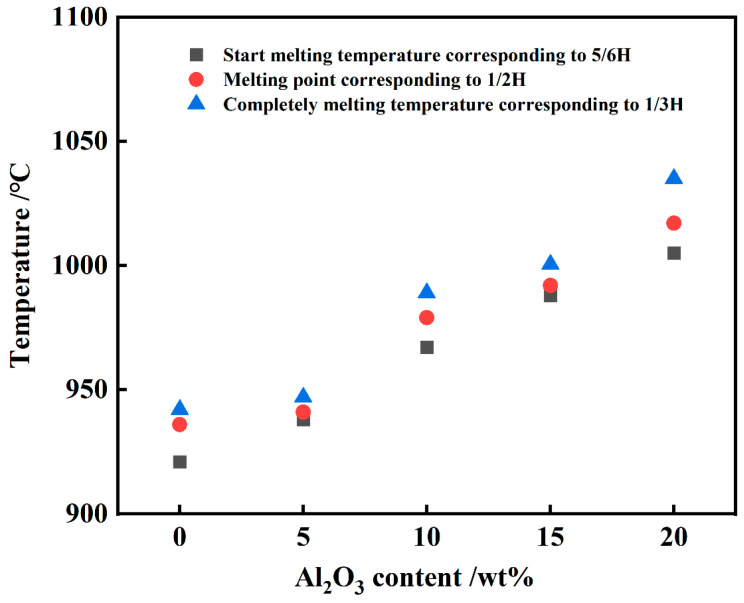
Effect of Al_2_O_3_ content on melting temperature of aluminum slag.

Figure 4 illustrates that as the slag spreads across the substrate, its height decreases while its diameter increases with temperature. Notably, there is significant variability in the slag’s morphological transformation across differing content ratios due to varying melting points. Liu et al. [29] emphasized that t the ratio of substance contents significantly influences the slag’s melting point. Consequently, it is essential to determine the melting temperatures of slags with varying Al_2_O_3_ concentrations. In this investigation, the hemispherical point temperature was designated as the slag melting point, with the temperature ranges corresponding to 5/6 and 1/3 of the slag’s original height (denoted by H) regarded as the melting temperature spectrum. Figure 5 illustrates the effect of Al_2_O_3_ content on the starting melting temperature, melting point, and complete melting temperature of slag. The figure indicates that slag with a higher Al_2_O_3_ content exhibits a higher melting point and a broader melting temperature range. This occurs because, firstly, Al_2_O_3_ is a substance with a high melting point, and increasing its content in the slag raises the melting point to a certain degree. Secondly, Al_2_O_3_ contributes to the formation of more aluminosilicates, which have high melting points, thereby continuously elevating the various temperatures of the slag during the wetting process.

### 3.2. The Apparent Contact Angle Between the Molten Slag and the SiC Substrate

**Figure 6:**
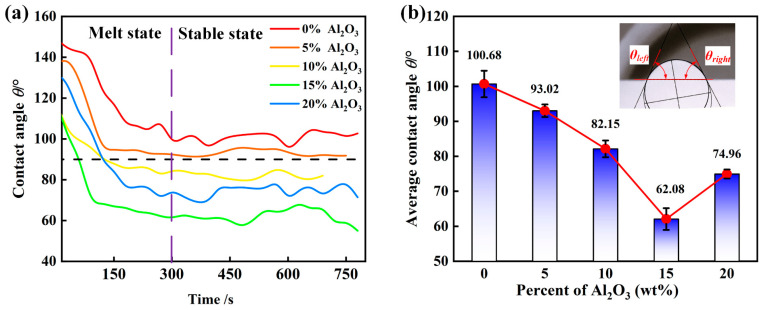
(**a**) Variation in the contact angle between slag and SiC substrate with holding time shows the trend; (**b**) Change of average contact angle with alumina content.

The apparent contact angle (*θ*) was determined from captured images. Subsequently, the left and right contact angles of the slag were computed using the ImageJ software, as illustrated in Figure 6a. The conclusive apparent contact angle represents the mean of the left and right contact angles observed in a particular frame [30]. Given that the consistency of the contact angle measurements can be influenced by various factors, it is essential to repeat each experiment on the SiC substrate a minimum of two times. When the difference in the measured apparent contact angles from the two trials falls within a defined range, the average value is accepted; however, if the difference is substantial, a third trial is necessary, and the average is calculated using the two experiments that have comparable apparent contact angles [31]. In this investigation, the onset of slag melting was designated as the reference point for changes in slag wetting, and the apparent contact angle at this moment was recorded as the initial contact angle (*θ_i_*) of the slag.

As illustrated in Figure 6b, an alteration in the contact angle is evident throughout the heating and holding processes, specifically between the Na_3_AlF_6_-Al_2_O_3_-CaF_2_ slag and the SiC substrate. Of particular note is the finding that in the final state, the contact angles between samples 3, 4, and 5 were significantly lower than the contact angles between samples 1 and 2, indicating that an increase in the aluminum oxide content facilitates the slag’s wetting of the refractory. As melting commenced in the slag samples, a notable decrease in the contact angle was observed with increasing temperature. With the prolongation of the holding time, the variation in the contact angle between the slag and SiC became less pronounced than during the temperature-increase phase, with the slag diffusing swiftly along the SiC substrate toward equilibrium. However, at the onset of the wetting reaction, the contact angle exhibited instability due to the gases produced by the interfacial reaction between the slag and the SiC refractory. These gases displaced the slag from the center. The contact angle was observed to reach a state of stability after 300 s, and an increase in the Al_2_O_3_ composition was observed, which increased the contact angle between the slag and the SiC substrate. After achieving a holding period of fifteen minutes, the final contact angles between slags 1# to 5# and the SiC substrate were documented as 102.7°, 91.8°, 82°, 55°, and 71.4°, correspondingly. It is noteworthy that the final contact angles decreased with increasing Al_2_O_3_ content. The experiment revealed that the slag exhibited effective wettability on an SiC substrate. Intriguingly, Slag 5# demonstrated a contact angle that was observed to exceed that of Slag 4#. This phenomenon can be attributed to the increased gas generation from the slag reaction as the temperature increases, along with the intensified volatilization of Na_3_AlF_6_. These factors hinder the complete melting and wetting of the substrate by slag. Following an extended hold period, during which the thermal history of the slag has influenced the interaction between the slag and the substrates, the contact angles are reduced, and the relationship stabilizes.

**Figure 7:**
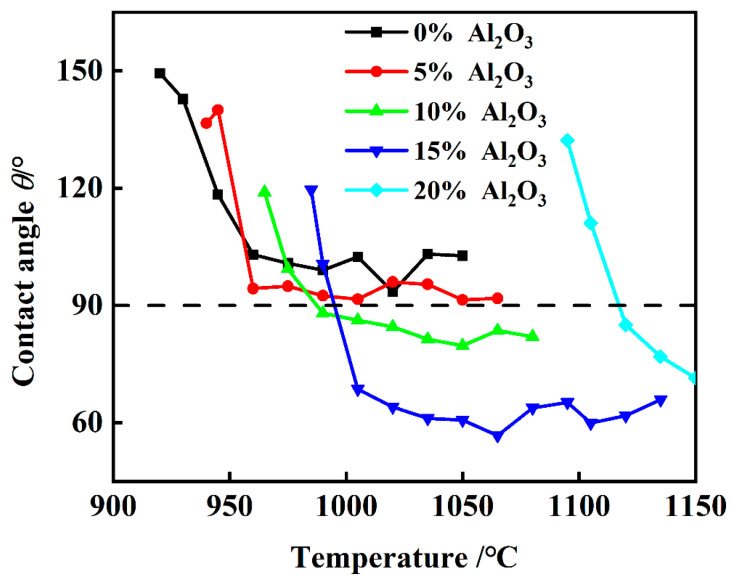
Variation in the contact angle between slag and the SiC substrate with temperature.

Figure 7 shows a visual representation of the variation in the contact angle between the aluminum slag and silicon carbide substrate as a function of increasing temperature. Typically, a contact angle of less than 90° indicates the transition of the slag and substrate into a wetting state. The 1# and 2# slags, with 0 wt% and 5 wt% Al_2_O_3_ content, exhibited contact angles of 102.44° and 91.6°, respectively, at 1000 °C, both of which remained greater than 90°, indicating that they did not wet the substrate throughout the heating process. In contrast, the 10 wt% (3# slag) and 15 wt% (4# slag) Al_2_O_3_ contents entered the wetting state with the SiC substrate before reaching 1000 °C. The 5# slag, containing 20 wt% Al_2_O_3_, did not melt at 1000 °C and thus failed to wet the SiC substrate. The 2# slag (5 wt% Al_2_O_3_) transitioned to wetting at approximately 950 °C, while the 3# slag (10 wt% Al_2_O_3_) reached this state around 980 °C, the 4# slag (15 wt% Al_2_O_3_) did so post-1000 °C, and the 5# slag (20 wt% Al_2_O_3_) at around 1120 °C, the earliest time at which it wetted the substrate. In this study, we report the contact angles of aluminum slag on SiC-based refractory materials, which are influenced by the Al_2_O_3_ content in the slag. We found that increasing the Al_2_O_3_ content leads to decreased contact angles, indicating improved wettability. However, discrepancies arise when comparing our results with those of studies that focus on different slag compositions or experimental setups. For instance, studies using higher Al_2_O_3_ concentrations (>10 wt%) report significantly different contact angles compared to industrial compositions (typically around 2.5 wt% Al_2_O_3_). This highlights the importance of using industrially relevant compositions in this study. Generally, the contact angle between the slag and the SiC substrate decreased with increasing Al_2_O_3_ content and temperature.

According to these results, the wettability of the Na_3_AlF_6_-Al_2_O_3_-CaF_2_ slag on the SiC substrate increases with an increase in the Al_2_O_3_ content and temperature. The analysis showed that during the temperature increase, high-melting-point phases containing aluminum were generated, leading to a surge in the melting point of the slag. The dissolution reactions of the Al_2_O_3_-containing slag and its continuous contact reaction with the SiC surface increased the mass transfer diffusion of molecules or ions in the slag, which led to the enhancement of the mobility of the slag droplets, thus allowing the slag to reach a wetting state.

### 3.3. Effect of Al_2_O_3_ Content on Surface Tension and Interfacial Properties Between Slag and Silicon Carbide Substrate

During the process of wetting, as the slag passes through the apertures on the refractory material’s surface, erosion occurs within the material caused by capillary forces. The driving force behind this phenomenon is related to the contact angle between the slag and the refractory material and the surface tension of the slag itself. The following formula expresses this relationship [32,33,34]:

(1) $$\Delta p=\left(2\gamma_{l}\cos\theta \right)/r$$

where Δp is the driving force of slag penetration (MPa), $\gamma_{l}$ is the surface tension of the slag (mN/m) [32,35], as detailed in Table 5. θ is the contact angle between the slag and the refractory substrate (°), and *r* is the radius of the capillary (mm):

The magnitude of the penetration pressure of slag with different Al_2_O_3_ contents on a SiC refractory substrate can be calculated using Equations (1) and (2). During the wetting process, the relationship between the contact angles of slag with different Al_2_O_3_ contents and the SiC refractory substrate was *θ*_0 wt%_ > *θ*_5 wt%_ > *θ*_10 wt%_ > *θ*_20 wt%_ > *θ*_15 wt%_. Furthermore, the relationship between the capillary pressure of slag with five different Al_2_O_3_ contents and the SiC refractory substrate can be calculated as Δ*p*_0 wt%_ < Δ*p_5_*_wt%_ < Δ*p*_10 wt%_ < Δ*p*_20 wt%_ < Δ*p*_15 wt%_. It can be observed that the slag with 15wt% Al_2_O_3_ content has the largest penetration drive and the strongest penetration ability on the SiC substrate. At high temperatures, the slag dissolution reaction produces a liquid phase that reacts with the refractory material, increasing the permeability of the slag and thus enhancing the slag driving force.

**Figure 8:**
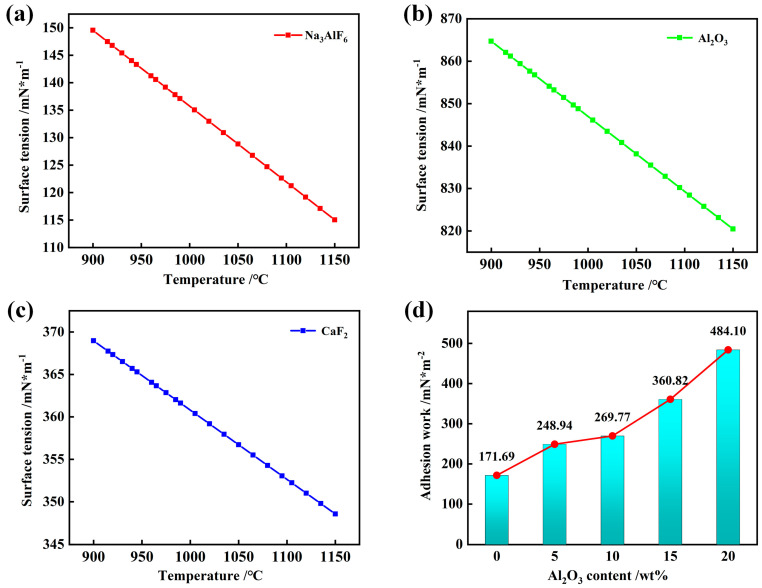
Effect of Al_2_O_3_ content on surface tension and adhesion work between aluminum slag and SiC substrate. (**a**–**c**) Variation of surface tension of each component of slag with temperature; (**d**) change in adhesion work with alumina content.

Figure 8a–c illustrates the surface tension of selected slags as a function of temperature and alumina (Al_2_O_3_) content. The surface tension exhibits a linear decrease with increasing temperature, which is in agreement with Schmetterer’s findings [37]. As the temperature increases, the thermal motion of the ions intensifies, leading to increased inter-particle spacing and, consequently, weaker interactions between the liquid particles and those at the liquid surface, thereby reducing the surface tension. Additionally, Figure 8a–c demonstrates the relationship between surface tension and Al_2_O_3_ content at various temperatures. At a constant temperature, the surface tension increases with increasing Al_2_O_3_ content. According to the ionic theory, the incremental addition of Al^3+^ cations disrupts oxygen bonds due to their strong electrostatic attraction. When slag contains moderate levels of Al_2_O_3_, the surrounding O^2−^ ions are attracted by Al^3+^ cations, reducing the O^2−^ accumulation on the surface and enhancing the surface tension of the molten slag [26]. Other studies focusing on the Al system have reported surface tension values that differ from our findings, primarily due to the unique surfactant behavior of alkali metals in liquid aluminum. This highlights the importance of considering specific slag compositions and experimental conditions when comparing the surface tension results.

The wetting and spreading of slag on the refractory surface are influenced by temperature, gravity, and adhesion force. The adhesion force, arising from molecular interactions between the liquid and solid, is a key factor affecting wettability. Adhesion work is defined as the energy required to disengage a liquid from a solid surface [38,39,40]. This concept was elucidated using the Young–Dupre equation, as shown in Equation (2). Figure 8d illustrates the influence of the Al_2_O_3_ content on the adhesion work between the slag and SiC. The Wad values for various systems indicate a rapid increase in adhesion work with increasing Al_2_O_3_ content in the slag. Specifically, the adhesion work of slag 1#, which contains 0% Al_2_O_3_, measures 171.68 mN/m^2^, whereas slag 5#, with a 20% Al_2_O_3_ content, exhibits an adhesion work of 484.09 mN/m^2^. This escalation is predominantly attributed to the enhanced wettability between the slag and the SiC surface, a factor heavily influenced by the Al_2_O_3_ content.

(2) $$W_{ad}=\gamma_{sg}+\gamma_{\mathrm{lg}}+\gamma_{sl}=\gamma_{\mathrm{lg}}\left(1+\cos\theta \right)\;\;0\leq \theta \leq 180{}^{\circ}$$

where $W_{ad}$ is the adhesion work of the slag and substrate, *θ* is the contact angle, and $\gamma_{\mathrm{lg}}$ is the surface tension coefficient.

In accordance with the established formula, the magnitude of the contact angle is inversely proportional to the degree of wettability. Consequently, as the contact angle increases, the adhesion force decreases, and the wettability deteriorates.

### 3.4. Analysis of the Microstructure and Interfacial Behavior of the Interface Between Slag and Refractory Materials

**Figure 9:**
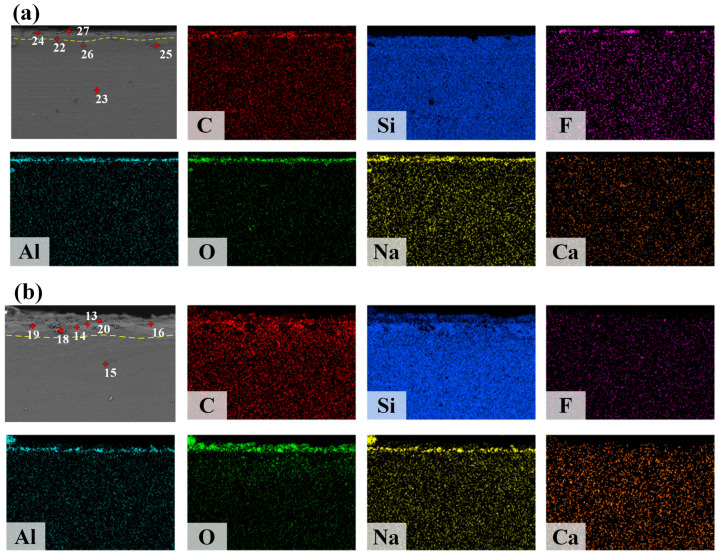
SEM images and elemental mapping across the interface between the slag and SiC, accompanied by the corresponding point-scanning results (as detailed in the table at the bottom). (**a**) 5 wt% Al_2_O_3_; (**b**) 20 wt% Al_2_O_3._

The chemical composition and elemental distribution of the slag-substrate interface were studied after the wetting experiments using SEM-EDS analysis. Figure 9 illustrates the elemental distribution at the interface between the slag droplets and the substrate for samples 2# and 5#. As shown in the figure, the microstructure can be categorized into two distinct layers: the slag interface layer and the original SiC substrate. The surface scanning results shown in the figure reveal the distribution of the major elements. From the first to the second layer, there is a gradual decrease in the contents of Al, O, and Na, while Si and C exhibit an opposite trend. Notably, the enriched regions of Al and O elements coincide, as do those of Al, Na, and O elements, as well as Si and C elements. Furthermore, the mass ratio of each component, as presented in Table 6, indicates that the primary phases within the substrate surface layer are Al_2_O_3_, Na_3_AlF_6_, and CaF_2_. By comparing the Si and C elements on the two substrate surfaces, it is observed that the Si element on the substrate surface of Slag 5# has obvious stratification and less content, and the C element is enriched more in the first layer. Physical and chemical dissolution occurs at the contact surface of Al_2_O_3_ and the electrolyte. The dissolved Al_2_O_3_ diffuses from the contact surface to the inside of the electrolyte, forming an adherent aluminum slag with the electrolyte. Aluminum and sodium phases will diffuse from the slag to the interfacial layer, while Al_2_O_3_ dissolves, and aluminum ions diffuse from the substrate to the interfacial layer. Both substrates comprise the Al-Na-F-O second phase in the SiC grains. The content of the second phase in Slag 5# is higher than that in Slag 2#. The interaction between the slag and substrate is limited due to the few reaction layers are detected at the interface between the slag and SiC refractory.

**Figure 10:**
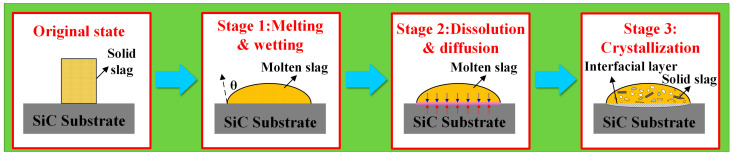
Adhesion mechanism of SiC substrate by Na_3_AlF_6_-Al_2_O_3_-CaF_2_ slag.

In summary, the adhesion mechanism of the specified molten slag to the SiC substrate is illustrated in Figure 10. During heating, solid slag melts into a liquid state, and the contact angle decreases. Initially, the high surface tension of the slag due to the presence of a solid phase hinders wetting and prevents SiC dissolution. As the temperature rises, the surface tension of the slag drops, reducing the contact angle to a stable level. This improved wetting facilitated the penetration of the SiC substrate. Once the slag was fully liquefied, the dissolution of Al_2_O_3_ accelerated. However, the rapid heating rate resulted in a shorter dissolution time. Simultaneously, fluoride and aluminum ions from the slag diffused into the SiC substrate and reacted with it. The dissolution of Al_2_O_3_ is another critical factor contributing to the adhesion of SiC. The distribution of Na, O, and Al from the surface to the substrate layer was evident in the scan results shown in Figure 9. The dissolution of Al_2_O_3_ and the reactions involving Na_3_AlF_6_, SiO_2_, and Al_2_O_3_ suggested the precipitation of high-melting-point phases in the interfacial layer. The products gradually covered the substrate beneath the droplet, preventing the further slag dissolution. After the wetting experiments, no significant adhesion was observed on the SiC substrate. This was due to the good wettability and dissolution of Al_2_O_3_, which contributed to adhesion issues between the slag and the SiC refractory.

## 4. Conclusions

This investigation explores the interfacial wetting and adhesion phenomena between Na_3_AlF_6_-Al_2_O_3_-CaF_2_ slag and SiC substrates through high-temperature wetting experiments. Key parameters pertinent to the wetting and spreading characteristics were meticulously measured and calculated to elucidate the influence of varying Al_2_O_3_ concentrations on the wetting behavior and adhesive attributes of slag droplets on SiC. The following conclusions were derived from our analysis:

(1) The experimental wetting process is summarized in three stages: melting and wetting, dissolution and diffusion, and crystallization. Two primary factors that influence substrate adhesion are considered: good wettability and the dissolution of Al_2_O_3_ in the slag. The interaction between the slag and the SiC substrate is described as non-reactive wetting, and the results demonstrate that the melting temperature of the Na_3_AlF_6_-Al_2_O_3_-CaF_2_ slag increases significantly with the addition of Al_2_O_3_. This increase in temperature leads to enhanced mobility of the elements within the slag, facilitating improved mass transfer and diffusion of molecules or ions. Furthermore, as the Al_2_O_3_ content increases, the contact angle between the cryolite-based molten salt and SiC decreases, reducing the height of the resulting slag layer and gradually increases the wetting radius, thus rendering the slag more effective at wetting and spreading on the substrate.
(2) Theoretical calculations indicate that Al_2_O_3_ enhances both surface tensions of the slag, thereby increasing the adhesion work between the slag and SiC. The initial spreading of the slag on the SiC substrate is primarily due to the reduction in the surface tension. The penetration depth of the slag into the SiC substrate gradually increased with increasing Al_2_O_3_ content, resulting in more severe penetration into the SiC refractory material. At high temperatures, the liquid slag phase formed within the refractory entered the SiC pores, promoting the penetration of the slag.
(3) In summary, this study provides a comprehensive analysis of the wettability and adhesion mechanisms of aluminum slag on SiC-based refractory materials. Increasing the Al_2_O_3_ content in the slag can reduce its wettability on SiC, thereby minimizing slag adherence to the ladle lining. Material Selection: Using SiC-based refractory materials with optimized Al_2_O_3_ content can improve the ladle lining’s resistance to slag penetration and mechanical erosion. Surface Treatment: Applying surface treatments or coatings that further reduce the wettability of the slag on SiC can enhance the durability of the ladle lining. These results have important implications for optimizing the design of vacuum ladles and other industrial equipment used in aluminum electrolysis. While our study provides valuable insights into the interactions between aluminum slag and SiC-based refractory materials, several avenues for future research remain. For instance, further investigations could explore the effects of other slag components, such as CaF_2_ and Na_3_AlF_6_, on wetting and adhesion behavior. Additionally, the impact of operational parameters, such as temperature and applied voltage, on these interactions can be studied in more detail. Long-term durability tests under realistic industrial conditions would also be beneficial for assessing the performance of optimized refractory materials over an extended period.

## Figures and Tables

**Table 1:** Chemical composition of the slag.

Chemical	Specification
**Na_3_AlF_6_**	≤0.2%
**Al_2_O_3_**	94 HRA
**CaF_2_**	0.14

**Table 2:** Composition ratio of slag samples (wt%).

Sample Slag	Na_3_AlF_6_	Al_2_O_3_	CaF_2_
1#	95	0	5
2#	90	5	5
3#	85	10	5
4#	80	15	5
5#	75	20	5

**Table 3:** Chemical composition of the SiC refractory substrate.

Component	SiC	SiO	Fe_2_O_3_	Free Silicon Content
**Content/mass%**	99.45	0.02	0.01	≤0.1

**Table 4:** Performance parameters of SiC refractory substrate.

Properties	Silicon Carbide Substrate
**Apparent porosity**	≤0.2%
**Rockwell hardness**	94 HRA
**Poisson’s ratio**	0.14

**Table 5:** Surface Tension of the Substances in the Slag [36].

Chemical	Surface Tension (mN/m)
**Na_3_AlF_6_**	273.74–0.138 T
**Al_2_O_3_**	1024–0.177 T
**CaF_2_**	442.4–0.0816 T

**Table 6:** Element contents of selected points in Figure 10 (wt%).

Sample	Point	Element Content/wt%						
		C	O	F	Na	Al	Si	Ca
5	22	41.47	4.81	0.31	0.34	0.34	52.61	0.12
	23	34.11	1.16	0.16	0.09	0	64.48	0
	24	48.22	23.24	1.43	2.93	7.88	15.13	1.17
	25	66.05	1.3	0.04	0.11	0	32.42	0.08
	26	37.54	1.22	0.17	0	0	61.03	0.03
	27	33.3	30.19	0.49	8.61	12.64	13.66	1.12
20	13	49.38	1.84	0.04	0	0	48.69	0.05
	14	35.24	1.29	0.09	0.01	0	63.37	0
	15	36.32	2.34	0.11	0.04	0	61.08	0.1
	16	29.74	1.73	0.19	0.06	0	68.1	0.19
	18	76.01	8.86	0.43	0.17	0	14.1	0.43
	19	50	1.88	0.09	0	0	47.95	0.08
	20	11.17	42.93	0.06	12.31	13.76	19.68	0.1

## Data Availability

The original contributions presented in this study are included in the article. Further inquiries can be directed to the corresponding authors.
